# SP1 induced long non-coding RNA LINC00958 overexpression facilitate cell proliferation, migration and invasion in lung adenocarcinoma via mediating miR-625-5p/CPSF7 axis

**DOI:** 10.1186/s12935-020-1099-0

**Published:** 2020-01-23

**Authors:** Longhai Yang, Lili Li, Zizi Zhou, Yi Liu, Jinyuan Sun, Xiaoming Zhang, Huiyu Pan, Song Liu

**Affiliations:** 1 Department of Cardiothoracic Surgery, Shenzhen University General Hospital/Shenzhen University Clinical Medical Academy, No. 1098 Xueyuan Road, Xili University Town, Shenzhen, 518055 Guangdong China; 2 Respiratory Medicine, Liaocheng People’s Hospital of Shandong Province, Liaocheng, 252000 Shandong China; 3 Department of Respiratory Medicine, Xinhua Hospital, School of Medicine, Shanghai Jiaotong University, No. 1665 Kongjiang Road, Yangpu District, Shanghai, 200092 China

**Keywords:** LINC00958, miR-625-5p, CPSF7, SP1, LAD

## Abstract

**Background:**

Increasing evidences have underlined the importance of long non-coding RNAs (lncRNAs) in human malignancies. LINC00958 has been found involved in some cancers. However, the underlying mechanical performance of LINC00958 in lung adenocarcinoma (LAD) has not been explored yet.

**Methods:**

The expression of relevant mRNA and protein were measured by qRT-PCR and western blot assays. EdU, colony formation, TUNEL and transwell assays were performed to investigate the function of LINC00958 on LAD progression. Luciferase reporter, RNA pull down and RIP assays were conducted to investigate the molecular mechanism of relevant RNAs.

**Results:**

LINC00958 was found notably overexpressed in LAD, which was associated with the stimulation of its promoter activity induced by SP1. LINC00958 depletion dramatically inhibited LAD cell proliferation, migration and invasion capacities by acting as a miR-625-5p sponge. MiR-625-5p curbed LAD progression via targeting CPSF7 and down-regulating its expression. Mechanically, LINC00958 was identified as a competing endogenous RNA (ceRNA) and positively regulated the expression of CPSF7 via sponging miR-625-5p.

**Conclusions:**

LINC00958 might drive LAD progression via mediating miR-625-5p/CPSF7 axis, indicating the potential of targeting LINC00958 for the treatment of LAD.
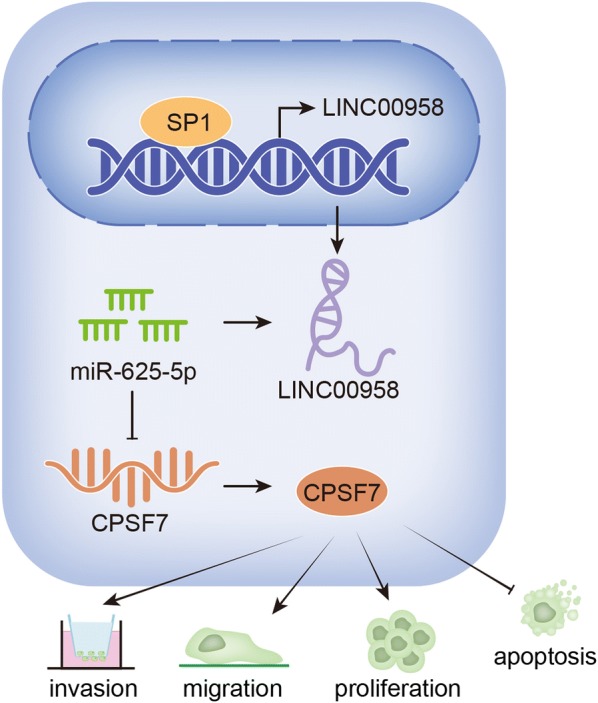

## Background

Lung cancer is a leading cause of cancer-associated deaths worldwide [1]. Non-small cell lung cancer (NSCLC) constitutes for approximately 85% of the diagnosed lung cancer cases [2]. Notably, lung adenocarcinoma (LAD) accounts for almost 50% of NSCLC cases, becoming the most predominant histological pathological subtype of lung cancer [3]. With steady rising morbidity and mortality rates, LAD is becoming a major threat for public health [4]. In the past decades, despite significant progress has been achieved in available therapeutic strategies, the 5-year overall survival rate for LAD patients remains unsatisfied, which is mainly attributed to local invasiveness and distant metastasis [5]. Unknown molecular events involved in facilitating LAD advancement needs to be explored. Revealing the underlying pathologic mechanism could help shed light on promising novel therapeutic targets for LAD.

Long non-coding RNAs (lncRNAs) are a group of transcripts with more than 200 nucleotides in length, yet without the potential to encode proteins [6]. Previous reporters have revealed that many lncRNAs were closely associated with a wide array of biological processes, such as cell proliferation, apoptosis, invasion, migration and metastasis [7–9]. LncRNAs have emerged as new key molecular regulators in the development and progression of various cancers due to its important role in pathologic progresses [10–12]. The aberrantly expressed lncRNAs might serve as diagnostic biomarker and treatment target for many kinds of cancers, including LAD [13].

Described as a group of highly conserved non-coding small RNAs, microRNAs (miRNAs) have a length of about 22 nucleotides and participate in a wide range of biological cellular courses of human diseases, including cancers [14, 15]. Muhammad et al. once revealed that miR-203 was expressed at high levels in breast cancer and indicated that anti-miR-203 might be used as a promising therapeutic target for the treatment of breast cancer [16]. Recently, the competing endogenous RNA (ceRNA) role of lncRNAs has received substantial attention in the domain of anti-cancer research. It is a new regulatory mechanism in which lncRNAs could mediate the targets of shared binding miRNAs, consequently imposing additional level of post-transcriptional regulation [17]. Previously, a collection of lncRNAs have been found aberrantly expressed in LAD [18]. LINC00958 has been found abnormally highly expressed and identified as a candidate oncogene in some cancers, such as bladder cancer and endometrial cancer [19, 20]. Furthermore, LINC00958 has been uncovered to regulate the behaviors of gliomagenesis via acting as a ceRNA to impact the expression and biological function of downstream miR-203/CDK2 [21]. However, the expression status, biological role and potential mechanical function of LINC00958 in LAD remain largely obscure.

This study was intended to uncover the important role and mechanical correlation of LINC00958 with LAD development and progression, aiming to hopefully provide a novel effective target for LAD treatment.

## Materials and methods

### Tissue collection

The paired tissue samples of LAD and corresponding normal tissues were collected from 64 patients with LAD between 2013 and 2018. The ethical approval was obtained from the Ethics Committee of Shenzhen University General Hospital. All the patients didn’t receive any treatment of chemotherapy or radiotherapy before this study. The collected tissue specimens were immediately frozen in liquid nitrogen at − 80 °C. Informed consents were signed by all the participants.

### Cell lines

Human lung adenocarcinoma (LAD) cell lines (H1975, PC9, A549 and Calu3) and normal pulmonic cell line (HBE) were procured from American Type Culture Collection (ATCC; Manassas, VA). Dulbecco’s Modified Essential Medium (DMEM; Gibco, Grand Island, NY) was commercially acquired for cell culture purposes under humidified atmosphere with 95% air/5% CO_2_ at 37 °C. 1% antibiotics (HyClone, Logan, UT) including streptomycin (100 μg/ml) and penicillin G (100 U/ml), as well as 10% fetal bovine serum (FBS, Gibco) served as the medium supplements. Medium was changed every 3rd day.

### Quantitative real-time polymerase chain reaction (qRT-PCR)

The total RNA samples were acquired by use of TRIZOL reagent (Invitrogen, Carlsbad, CA) from cultured cell lines. The synthesis of complementary DNA (cDNA) was then achieved as per the guidebook of reverse transcription kit (Takara, Kyoto, Japan). For the quantitative analysis, SYBR Green PCR Master Mix (Takara) was used on the Step-One Plus Real-Time PCR System (Applied Biosystems, Foster City, CA). The thermal cycling condition was listed as below: pre-denaturation at 95 °C for 10 min, 40 cycles of denaturation at 95 °C for 15 s, annealing at 60 °C for 1 min, followed by extension at 72 °C for 30 s. The comparative 2^−ΔΔCt^ method was applied for calculation of relative gene expression level. The glyceraldehyde-3-phosphate dehydrogenase (GAPDH) gene or U6 small nuclear RNA was regarded as the internal control. The specific primer sequences were listed as below: Forward Primer for LINC00958, 5′-GTCTCCCTGGTTTCTCACAGTT-3′, Reverse Primer for LINC00958, 5′-TCCCTGGCTACAAATAACCACA-3′; Forward Primer for SP1, 5′-TGGCAGCAGTACCAATGGC-3′, Reverse Primer for SP1, 5′-CCAGGTAGTCCTGTCAGAACTT-3′; Forward Primer for CPSF7, 5′-ACAACAAGACCCCTGCAATTC-3′, Reverse Primer for CPSF7, 5′-ACTCCACCACATCATAGACTCC-3′; Forward Primer for miR-625-5p, 5′-GTAGAGGGATGAGGGGGAA-3′, Reverse Primer for miR-625-5p, 5′-CTCTACAGCTATATTGCCAGCCA-3′; Forward Primer for U6, 5′-ACCGTCAGCGAATCCTCTTC-3′, Reverse Primer for U6, 5′-AACAGGCTCGTGAAAGACCG-3′; Forward Primer for GAPDH, 5′-CTGGGCTACACTGAGCACC-3′, Reverse Primer for GAPDH, 5′-AAGTGGTCGTTGAGGGCAATG.

### Cell transfection

The duplex LINC00958-specific short hairpin RNAs (shRNAs) and negative control (NC) shRNAs (sh-NC) were constructed and procured from RiboBio (Guangzhou, China). To overexpress SP1 and CPSF7, the whole sequences of SP1 or CPSF7 was inserted into the pcDNA3.1 vector (RiboBio), termed pcDNA3.1/SP1 and pcDNA3.1/CPSF7. The empty pcDNA3.1 vector was seen as the negative control, termed pcDNA3.1. In addition, the miR-625-5p mimics and NC mimics, miR-625-5p inhibitor and NC inhibitor, were also designed by RiboBio. The 48 h of transfection in PC9 and A549 cells was implemented by using Lipofectamine 2000 (Invitrogen). The transfection efficiency of overexpression or knockdown was finally estimated by qRT-PCR assay.

### Colony formation

After transfection, the cultured cells of PC9 and A549 at logarithmic growth phase were trypsinized, reaped and re-suspended, then plated into the 6-well culture plate with the cell density of 500/well under the condition of 37 °C and 5% CO_2_. Following the 14-day cell culture process, the medium was replaced on the day 7. Clonogenic cells were then fixed by methanol for 20 min before staining with 0.5% crystal violet for 30 min. After that, clones were rinsed in phosphate buffer saline (PBS), and counted manually.

### EdU incorporation assay

Cells of PC9 and A549 after indicated transfection were reaped and cultivated in the 96-well culture plate at the cell density of 8000/well. Then cells were cultured with 25 μM of EdU medium diluent for 4 h, then fixed for 30 min by 4% paraformaldehyde and permeabilized for 10 min by 0.5% Triton X-100, following 1 × Apollo^®^ 488 staining for 30 min. Proliferating LAD cells were monitored as per the instruction of EdU incorporation assay kit from Ribobio, Cell nuclei were counterstained with the DAPI (Beyotime, Shanghai, China) in the darkroom for observation by fluorescent microscope (Leica, Wetzlar, Germany). Results were shown as the ratio of EdU-positive cells to DAPI-positive cells.

### TUNEL assay

Cells of PC9 and A549 were first seeded on coverslips for 10 min of fixation with 4% paraformaldehyde at room temperature, then rinsed in ice-cold PBS. After permeabilizing in 0.2% Triton X-100 for 15 min, cell apoptosis was assayed employing the One-Step TUNEL Apoptosis Assay Kit (Beyotime) in line with the user manual. PC9 and A549 cells were assessed by fluorescent microscope after DAPI staining. TUNEL-positive cells were determined using Image J software (National Institutes of Health, Bethesda, MD). Results were expressed as the ratio of TUNEL-positive cells to DAPI-positive cells.

### Transwell assay

The 8-mm pore size Transwell chambers (Millipore, Billerica, MA) was pre-coated with Matrigel membrane (Clontech, Madison, WI) for invasion analysis or without Matrigel membrane for migration analysis. After seeding transfected cells of PC9 and A549 (1 × 10^5^) into the upper chamber, the DMEM medium adding 10% FBS was prepared to supplement lower chamber. Following 24 h of cell culture, cells in the upper chamber were first removed by cotton swabs, while the invading or migrating LAD cells to the bottom were stained in 0.5% crystal violet solution after fixing in 4% paraformaldehyde. Five fields were randomly counted in each well under the microscope (magnification, ×200).

### Western blot

After culturing cells of PC9 and A549 in radio-immunoprecipitation assay (RIPA) buffer, the cell protein extracts were collected for separation by electrophoresis on the 10% sodium dodecyl sulfate polyacrylamide gel electrophoresis (SDS-PAGE). Cell protein samples were electro-transferred onto the polyvinylidene difluoride (PVDF) membranes (Millipore). Then, membranes were sealed with 5% nonfat milk and cultivated at 4 °C all night with the primary antibodies against E-cadherin (1: 50 dilution; Mouse monoclonal; ab1416), N-cadherin (1: 2000 dilution; Mouse monoclonal; ab98952), Vimentin (1: 1000 dilution; Mouse monoclonal; ab8978), slug (1: 1000 dilution; Rabbit polyclonal; ab27568), Cyclin D1 (1: 200 dilution; Rabbit monoclonal; ab16663), CDK4 (1: 2000 dilution; Rabbit monoclonal; ab199728), cleaved-caspase-3 (1: 500 dilution; Rabbit polyclonal; ab49822), caspase-3 (1: 500 dilution; Rabbit polyclonal; ab44976), PARP (1: 2000 dilution; Rabbit monoclonal; ab227244), CPSF7 (1: 2000 dilution; Rabbit monoclonal; ab131245) and GAPDH (1: 10,000 dilution; Mouse monoclonal; ab125247) as loading control. Samples were then rinsed thrice in tris-buffered saline-tween (TBST), and probed at room temperature for 2 h with their relative horseradish peroxidase (HRP)-tagged secondary antibodies (1: 10,000 dilution; Goat polyclonal; ab6789). All antibodies were procured from Abcam (Cambridge, MA). Samples were finally analyzed by the enhanced chemiluminescence (ECL) detection system (Millipore). Signals were all documented by the Gel Imagine System (Bio-Rad, Hercules, CA), and the gray value was compared using Image J software.

### Chromatin immunoprecipitation (ChIP)

The binding of SP1 to LINC00958 promoter was confirmed in line with the protocol of EZ-Magna ChIP KIT (Millipore). 4% paraformaldehyde was employed to fix cell samples of PC9 and A549 for 10 min for facilitating the DNA and protein cross-linking. The DNA was then randomly fragmented to 200–1000 base pairs (bp) by ultrasonic. Anti-SP1 antibody and control normal mouse anti-IgG antibody (Millipore) were used for immunoprecipitation with cross-linked chromatin DNA. Following washing and de-cross-linking, qRT-PCR was applied for analyzing the precipitates.

### Dual-luciferase reporter assays

For gene promoter analysis, the LINC00958 promoter after PCR amplification was inserted into the downstream of the firefly luciferase gene in pGL3-Basic vector (Promega, Madison, WI) to construct pGL3-LINC00958 promoter reporter vector. Then, the construct was co-transfected with pcDNA3.1/SP1 or pcDNA3.1 to HEK-293T cells (ATCC), as well as Renilla luciferase gene. Besides, the wild-type (WT) and mutated (MUT) miR-625-5p interacting sites in LINC00958 sequence or CPSF7 3′-UTR were acquired and inserted to the firefly luciferase gene in pmirGLO vector (Promega) for forming the LINC00958 WT/MUT and CPSF7 WT/MUT reporter vectors. Then, the constructs were co-transfected with miR-625-5p mimics or NC mimics to HEK-293T cells. Dual-Luciferase Reporter Assay System (Promega) was used 48 h after transfection. The relative luciferase activity was estimated using the Renilla luciferase and firefly luciferase.

### Subcellular fractionation

The subcellular fractionation assay in LAD cells of PC9 and A549 was implemented according to the user guide of PARIS™ Kit (Invitrogen). 1 × 10^6^ LAD cells were prepared and lysed in the cell fractionation buffer, then centrifuged for separating the fractions of cell nucleus and cell cytoplasm. The supernatant was collected as cell cytoplasm and transferred into fresh RNase-free tube. After washing again in cell fractionation buffer, the remaining lysates were centrifuged and cultivated in the cell disruption buffer. RNA extraction was conducted before RNA purification. The levels of LINC00958, GAPDH (indicator of cell cytoplasm) and U6 (indicator of cell nucleus) were tested by qRT-PCR.

### Fluorescence in situ hybridization (FISH) assay

The specific FISH probe to LINC00958 was synthesized by Ribobio. LAD cells of PC9 and A549 fixed in 4% paraformaldehyde for 20 min, then cultured with protease K at 37 °C for 10 min and rinsed in PBS. After dehydration in ethanol and denaturation, cells were incubated in hybridization solution (18 μl of prepared prer-hybridization solution and 2 μl of FISH probe) all night at 42 °C. Cell samples were then rinsed in 2 × saline sodium citrate (SSC)/25% formamide, stained with Hoechst solution for nuclear counterstaining, finally imaged by microscope.

### RNA pull down

The interaction between LINC00958 and miR-625-5p was examined using RNA pull-down assay by use of Pierce Magnetic RNA-Protein Pull-Down Kit (Thermo Fisher Scientific, Waltham, MA) as per manual. The miR-625-5p target sequences (wild type or mutant) were synthesized and Biotin-labeled into Biotin LINC00958 WT/MUT probes. Then, 50 pmol of probes were separately mixed with the protein extracts from LAD cells of PC9 and A549, and the 50 μl of magnetic beads for 1 h. The pulled-down mixture was eluted and assayed by qRT-PCR.

### RNA immunoprecipitation (RIP)

RIP assay was used to assay the relationships among LINC00958, CPSF7 and miR-625-5p with the application of EZ-Magna RIP RNA Binding Protein Immunoprecipitation Kit (Millipore). The cultured LAD cells of PC9 and A549 were first lysed in RIP lysis buffer, then collected and mixed with the RIP buffer containing magnetic beads and antibodies against human Ago2 or control IgG (Millipore). At length, the immunoprecipitated RNAs after purification was subjected to qRT-PCR.

### Flow cytometry for cycle analysis

Flow cytometry (BD Biosciences, USA) was adopted for cell cycle analysis. The transfected PC9 or A549 cells were cultivated and fixed with 500 μl of 70% cold ethanol. Afterwards, propidium iodide (PI, 400 μl) staining (Beyotime, Shanghai, China) were conduct. After 30 min of incubation at 4 °C, transfected cells were subjected to flow cytometer (FACScan, BD Biosciences, USA) to analyze the data.

### Animal study

Six male BALB/c nude mice (18–20 g, 6-week-old) were procured from Shi Laike Company (Shanghai, China) and employed with the ethical approval from the Institutional Animal Care and Use Committee of Shenzhen University General Hospital. The nude mice housed under special pathogen-free (SPF)-condition were injected subcutaneously with 1 × 10^6^ transfected PC9 cells, with tumor volumes examined every 4 days and calculated as 1/2 length × width^2^. Tumor samples were acquired from mice for weight assessment after 28 days of injection was terminated.

### Immunohistochemistry (IHC) assay

Tumors were obtained from the in vivo assay, followed by being fixed in paraformaldehyde. And then these samples were dehydrated in ethanol solutions, inset in paraffin and cut into 4-μm thickness. Subsequently, these sections were cultured with primary antibodies against Ki67, PCNA, E-cadherin and N-cadherin at 4 °C for all night. Afterwards, they were cultivated with HRP-conjugated secondary antibodies. All these sections were finally visualized through microscope (Olympus).

### Statistical analysis

All measurement data were given as the mean ± standard deviation (S.D.) from the three or more independent bio-repeats. Student’s test, one-way or two-way analysis of variance (ANOVA) was applied for statistical analyses with the help of PRISM 6 (GraphPad, San Diego, CA), with p-value less than 0.05 as threshold.

## Results

### LINC00958 is predominantly up-regulated in LAD and silencing LINC00958 suppresses LAD cell proliferation, migration and invasion

**Fig. 1:**
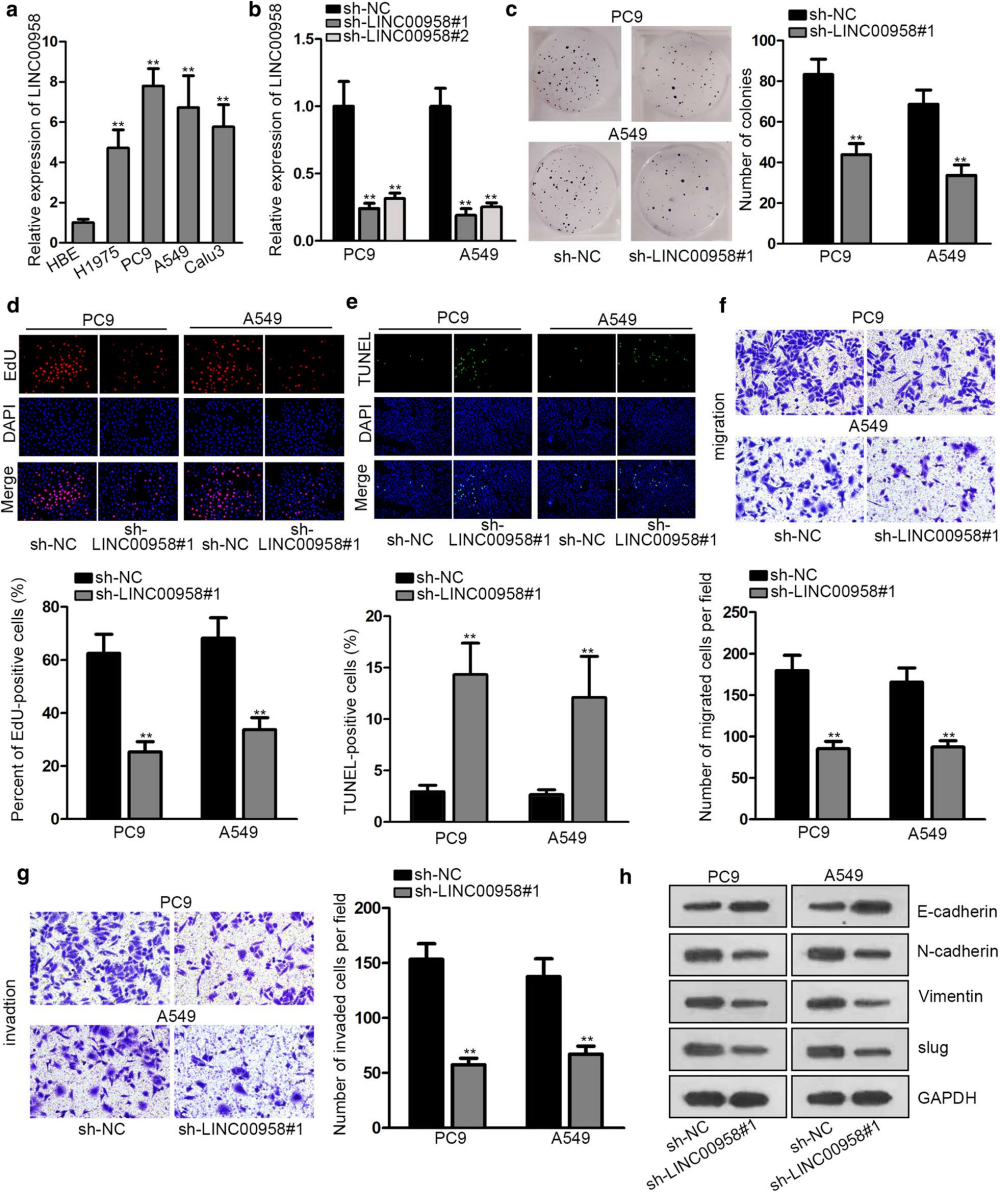
LINC00958 is predominantly up-regulated in LAD and silencing LINC00958 suppresses LAD cell proliferation, migration and invasion. **a** QRT-PCR analysis was used to determine the expression status of LINC00958 in LAD cell lines and normal pulmonic cell line (HBE). **b** The inhibitory efficiency of sh-LINC00958#1/2 was detected by qRT-PCR analysis. **c**, **d** Colony formation and EdU assays were performed to determine the proliferation ability of transfected cells. **e** TUNEL assay was used to evaluate cell apoptosis in different groups. **f**, **g** Transwell migration and invasion assays were conducted to assess the abilities of transfected cells to migrate and invade. **h** Western blot assay was performed to measure EMT-related protein expression level change in different groups. **P < 0.01

We investigated the expression status of LINC00958 in LAD tissues and cell lines firstly. Through qRT-PCR analysis, we observed that LINC00958 expression was higher in LAD tissues in contrast to adjacent non-tumor tissues (Additional file 1: Figure S1A). LINC00958 is predominantly overexpressed in LAD cell lines (H1975, PC9, A549 and Calu3) compared with normal pulmonary cell line (HBE) (Fig. 1a). To determine the biological function of LINC00958 in LAD cells, we used two sh-RNAs targeting LINC00958 to knockdown LINC00958 expression in PC9 and A549 cells. We selected sh-LINC00958#1 for subsequent studies for its better inhibitory efficiency (Fig. 1b). Colony formation assay showed that silencing LINC00958 significantly diminished the number of colonies (Fig. 1c). EdU assay results further proved that silencing LINC00958 dramatically weakened proliferation ability (Fig. 1d). Data from flow cytometry analysis of cell cycle revealed that LINC00958 depletion elevated the distribution at G1/G0 phase, resulting in cycle arrest at G0/G1 phase (Additional file 1: Figure S1B). After LINC00958 expression was decreased in PC9 and A549 cells, the expression of cycle-related proteins (cyclin D1, CDK4) was reduced and the expression of apoptosis-associated proteins (cleaved caspase-3, PARP) was increased, indicating that silenced LINC00958 could inhibit cell cycle and promote cell apoptosis (Additional file 1: Figure S1C). Moreover, TUNEL assay further indicated that LINC00958 knockdown could enhance cell apoptosis ability (Fig. 1e). In addition, transwell migration and invasion assays suggested that downregulation of LINC00958 significantly curbed LAD cells migration and invasion respectively (Fig. 1f, g). The expression alternation of EMT-related proteins further validated the anti-EMT role of LINC00958 knockdown in PC9 and A549 cells (Fig. 1h). Taken together, LINC00958 was highly expressed in LAD and LINC00958 knockdown restrained LAD cell proliferation, migration, invasion and EMT process.

### SP1 induces LINC00958 overexpression by serving as a transcription activator

**Fig. 2:**
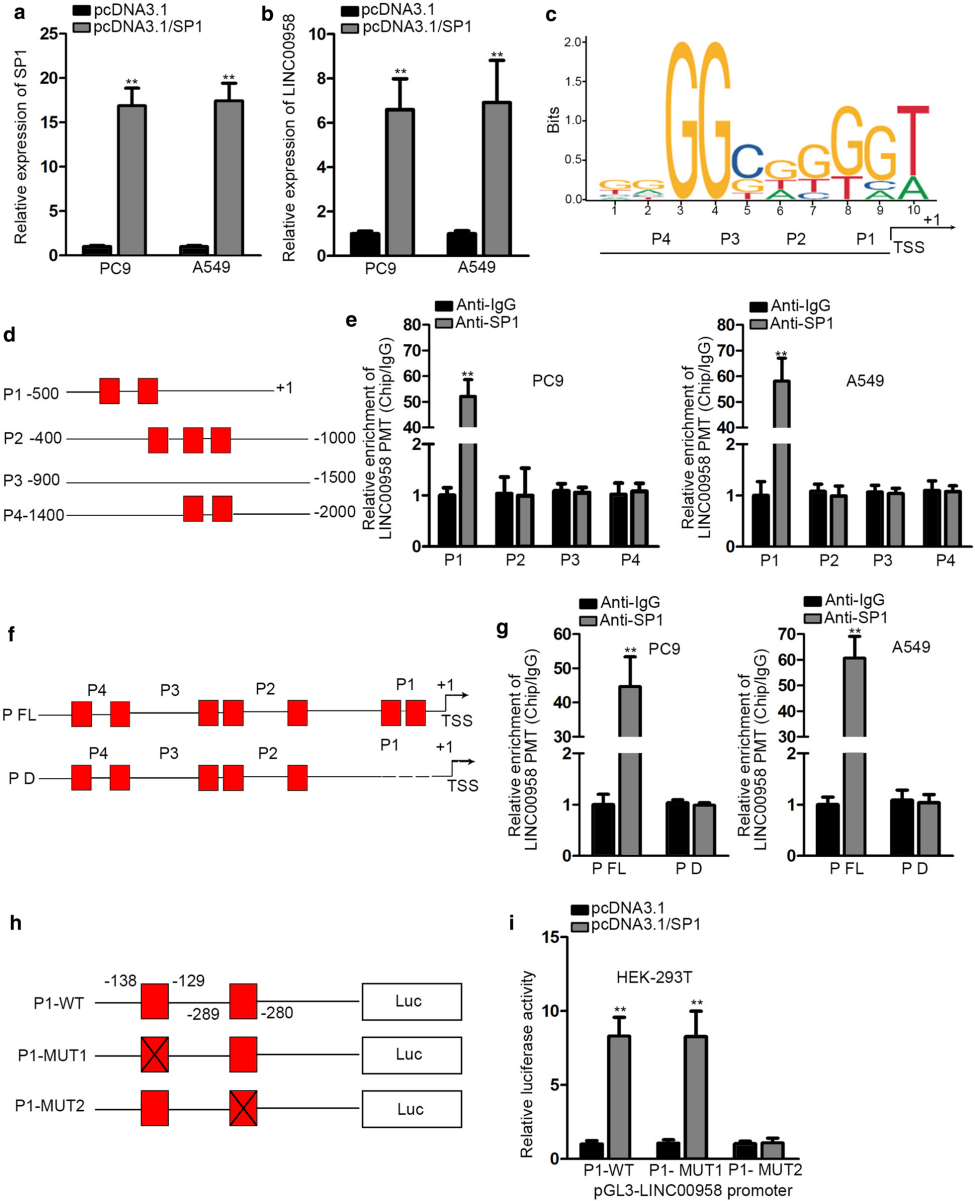
SP1 induces LINC00958 overexpression by serving as a transcription activator. **a** QRT-PCR analysis was performed to investigate the expression of SP1 after transfecting with pcDNA3.1 and pcDNA3.1/SP1. **b** QRT-PCR was performed to study the effect of pcDNA3.1/SP1 on LINC00958 expression. **c** The putative SP1 binding motif in − 2000 bp of human LINC00958 promoter. **d** LINC00958 promoter was divided into four sectional fragments. **e** ChIP assay using antibody targeting SP1 and IgG was performed to detect the affinity of SP1 with LINC00958 promoter. **f** The full LINC00958 promoter (P FL) and LINC00958 P1 deleted (P D) was constructed. **g** ChIP assay using antibody targeting SP1 and IgG was performed to determine the relative enrichment of LINC00958 promoter. **h** pGL3-P1-WT, pGL3-P1-MUT1 and pGL3-P1-MUT2 luciferase vectors were constructed. **i** HEK-293T cells were transfected with pcDNA3.1 and pcDNA3.1/SP1 and luciferase reporter assays was performed among P1-WT, P1-MUT1 and P1-MUT2. **P < 0.01

We preliminarily hypothesized that transcription factor might played a role in the aberrant up-regulation of LINC00958 in LAD. Using UCSC (http://genome.ucsc.edu/) and JASPAR (http://jaspardev.genereg.net/) databases, we found that SP1 might be a candidate transcription factor capable of binding to LINC00958 promoter region. Given the dual role of SP1 in the transcription activation of lncRNAs, we firstly performed qRT-PCR to investigate the effects of SP1 on LINC00958. We guaranteed the forced overexpression efficiency of pcDNA3.1/SP1 in the first place (Fig. 2a). Data from qRT-PCR indicated that LINC00958 expression was significantly up-regulated by overexpressing SP1 (Fig. 2b). Afterwards, SP1 expression was uncovered to be markedly upregulated in LAD tissues and cells (Additional file 1: Figure S1D, E). We then detected the DNA motif of SP1 in the promoter region of LINC00958 by JASPAR (Fig. 2c). Subsequently, we divided the promoter region into four sectional regions based on the putative binding sites of SP1 in LINC00958 promoter (Fig. 2d). To find out which sectional region in the LINC00958 promoter region actually bind to SP1, ChIP assay was conducted. ChIP data demonstrated that SP1 could bind to P1 sectional area (Fig. 2e). To further certify this observation, we constructed pGL3-LINC00958 promoter full region (P FL) and pGL3-LINC00958 promoter P1 deleted region (P D) (Fig. 2f). ChIP data showed that the deletion of P1 manifested barely no LINC00958 promoter enrichment change in anti-SP1 group, which indicating that P1 sectional region was crucial for the binding to SP1 (Fig. 2g). SP1 bind to LINC00958 promoter at about -500 to + 1 bp upstream transcription start site (TSS). Since JASPAR detected two specific binding sites (− 138 to − 129 and − 289 to − 280) in P1 fragment, we aimed to determine which precise sequence in P1 bind to SP1 subsequently. We constructed P1-WT, P1-MUT1 and P1-MUT2 for the purpose of luciferase reporter assay (Fig. 2h). After co-transfecting with pcDNA3.1/SP1, the promoter activity of P1-WT and P1-MUT1 increased significantly, while almost no luciferase activity variation in P1-MUT2 group which lacked the sequence from − 289 to − 280 (Fig. 2i). These data revealed that SP1 activated LINC00958 transcription in LAD via binding to LINC00958 promoter region at − 289 to − 280 site upstream TSS.

### LINC00958 directly interacts with miR-625-5p and miR-625-5p inhibition counteracts the effects of silencing LINC00958 on LAD progression

**Fig. 3:**
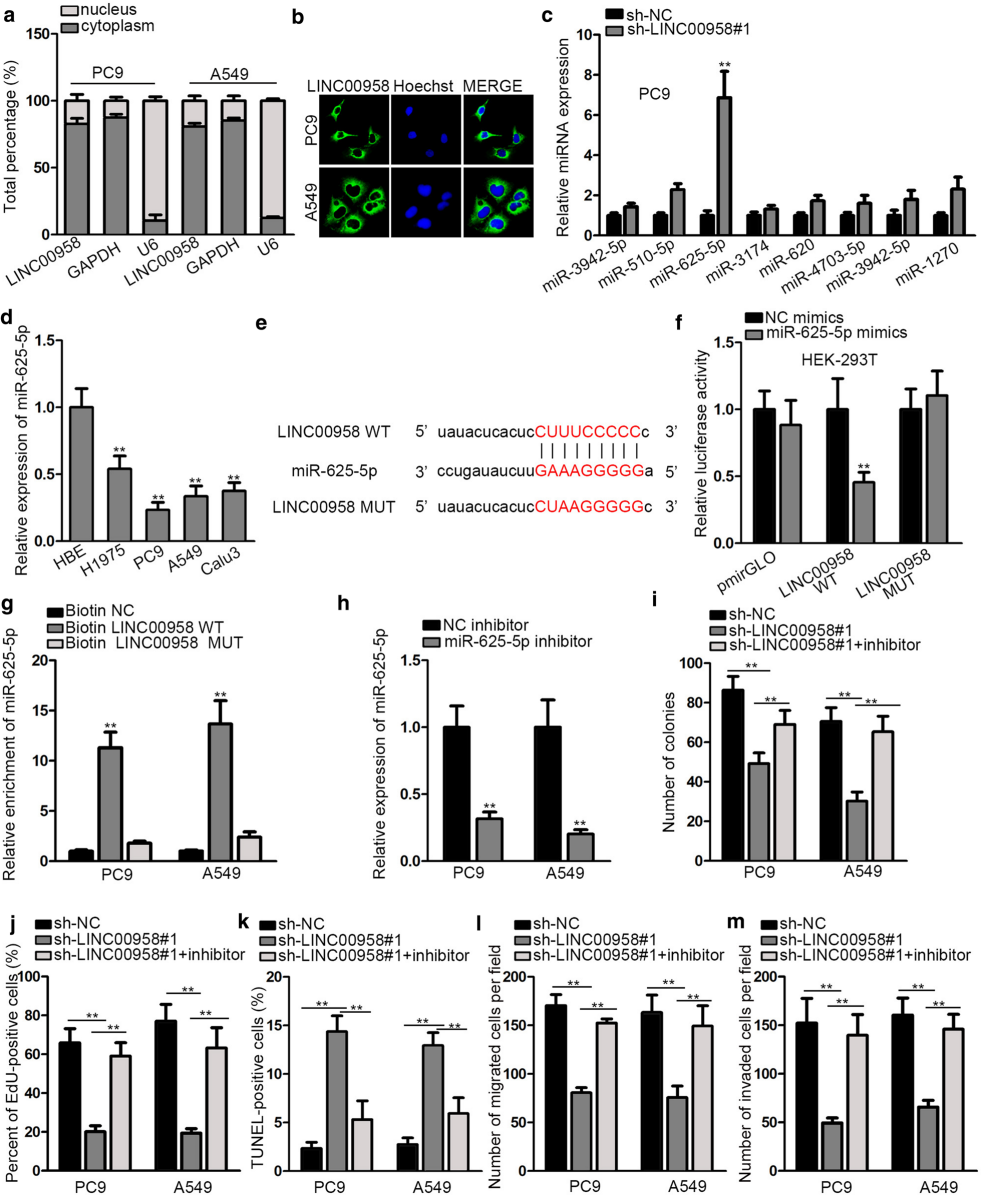
LINC00958 directly interacts with miR-625-5p and miR-625-5p inhibition counteracts the effects of silencing LINC00958 on LAD progression. **a**, **b** Subcellular fractionation and FISH assays were used to determine the situation of LINC00958. **c** QRT-PCR analysis was performed to determine candidate miRNAs expression after silencing LINC00958. **d** QRT-PCR assay was performed to detect the expression of miR-625-5p in LAD cell lines and normal pulmonic cell line. **e** Potential miR-625-5p binding site with LINC00958 detected by starBase. **f** Luciferase reporter assay found that miR-625-5p mimics could attenuate the luciferase activity of LINC00958-WT. **g** RNA pull down showed enriched miR-625-5p by biotin-labeled LINC00958-WT. **h** The inhibitory efficiency of miR-625-5p inhibitor was examined by qRT-PCR. **i**–**m** A list of functional experiments was conducted in PC9 and A549 cell lines in a rescue manner. **P < 0.01

Depending on the sub-cellular location, lncRNAs exerted different biological role. We found that LINC00958 was predominantly situated in the cytoplasm of PC9 and A5489 cells from subcellular fractionation and FISH assays results (Fig. 3a, b). Utilizing starBase database (http://starbase.sysu.edu.cn/), we found eight potential combinable miRNAs after limiting binding conditions (Clip data: medium stringency ≥ 2; Degradome data: with or without data; Pan-cancer: with or without data). Through qRT-PCR analysis, we noticed that only miR-625-5p expression was dramatically elevated in the presence of sh-LINC00958#1 in comparison of other miRNA candidates (Fig. 3c). In addition, we examined the aberrant down-regulation of miR-625-5p in LAD tissues and cells (Additional file 2: Figure S2A, Fig. 3d). Subsequently, miR-625-5p putative binding site in the sequence of LINC00958 was obtained by starBase bioinformatics analysis (Fig. 3e). After co-transfecting miR-625-5p mimics with luciferase vector containing LINC00958-WT or LINC00958-MUT, we found that the luciferase activity of LINC00958-WT was significantly decreased in HEK-293T cells, while no change in that of LINC00958-MUT (Fig. 3f). RNA pull down assay was performed to further confirm the interaction. It manifested that miR-625-5p was significantly enriched by biotinylated LINC00958-WT (Fig. 3g). These findings revealed that LINC00958 functioned as a miR-625-5p sponge in LAD. To further verify whether LINC00958 facilitated LAD progression via sponging miR-625-5p, we conducted a collection of rescue experiments. We forced the down-regulation of miR-625-5p by transfecting miR-625-5p inhibitor plasmid (Fig. 3h). We found that miR-625-5p inhibitor could significantly counteract the inhibitory effects of LINC00958 on LAD cells proliferation (Fig. 3i, j). Moreover, data from flow cytometry, western blot and TUNEL assays demonstrated that miR-625-5p inhibition could rescue the promoting effect of LINC00958 depletion on cell cycle and apoptosis (Additional file 2: Figure S2B-C, Fig. 3k). Further, the attenuated capabilities of cell migration and invasion induced by silenced LINC00958 could be restored by inhibiting miR-625-5p expression (Fig. 3l, m, Additional file 2: Figure S2D, E). Briefly, LINC00958 directly targeted miR-625-5p and miR-625-5p inhibition could rescue the effects of LINC00958 downregulation on LAD progression.

### CPSF7 is identified the molecular target of miR-625-5p and is regulated by LINC00958

**Fig. 4:**
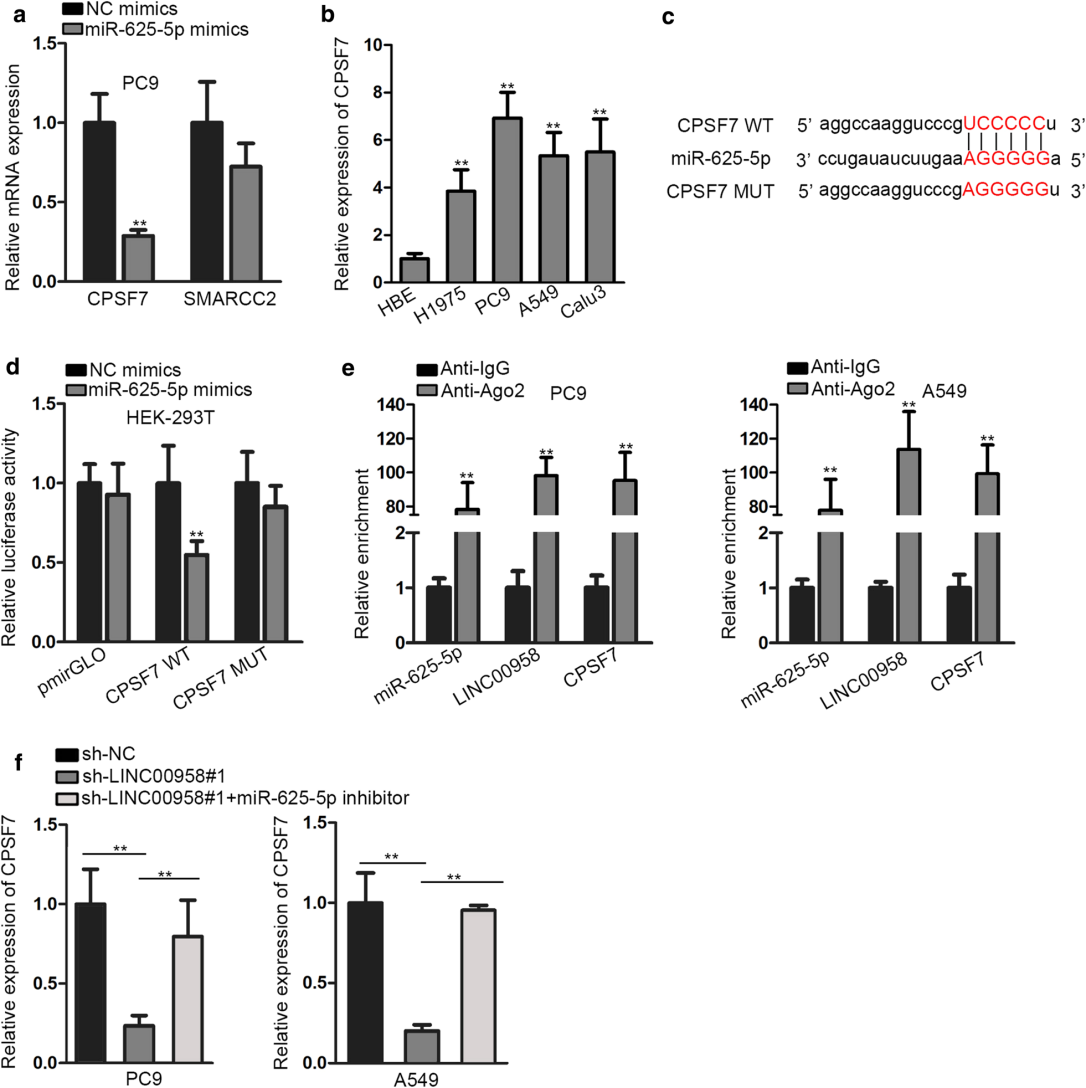
CPSF7 is identified the molecular target of miR-625-5p and is regulated by LINC00958. **a** QRT-PCR was performed to detect the expression of target genes after transfecting with miR-625-5p mimics. **b** The expression profile of CPSF7 in LAD cell lines and normal pulmonic cell line was detected by qRT-PCR. **c** Putative miR-625-5p binding sites in 3′UTR sequence of CPSF7 on starBase. **d** Luciferase reporter assay was performed to determine the association between miR-625-5p and CPSF7. **e** RIP was used to verify the interaction of miR-625-5p and LINC00958, as well as miR-625-5p and CPSF7. **f** QRT-PCR was performed to study the effect of miR-625-5p on LINC00958 mediated CPSF7. **P < 0.01

As largely documented, lncRNA can indirectly regulate the expression of specific mRNA by sponging miRNA. Herein, we used starBase to find out downstream targets of miR-625-5p. We screened out two target genes after restricting bind circumstance (Clip data: strict stringency ≥ 3; Degradome data: high stringency ≥ 3; Program Number: four). Through qRT-PCR and western blot analyses, we noticed that the expression of CPSF7 decreased most notably in PC9 cells after transfecting with miR-625-5p mimics compared with that of another candidate, SMARCC2 (Fig. 4a, Additional file 3: Figure S3A). Besides, CPSF7 expression was up-regulated in LAD tissues and cell lines, which was contrary to the expression pattern of miR-625-5p in LAD (Additional file 3: Figure S3B, Fig. 4b). In addition, the protein expression of CPSF7 in LAD cell lines was markedly elevated in contrast to HBE cells (Additional file 3: Figure S3C). Using starBase, we found miR-625-5p putative binding site in the 3′UTR sequence of CPSF7 (Fig. 4c). We then performed luciferase reporter assay to investigate the association between miR-625-5p and CPSF7. We observed that the luciferase activity of CPSF7-WT was dramatically impaired by miR-625-5p mimics, but not that of CPSF7-MUT group (Fig. 4d). RIP assays were performed to further confirm such physical interaction. The result uncovered the significantly enriched miR-625-5p, LINC00958, as well as CPSF7 in anti-Ago2 group in contrast to IgG control group (Fig. 4e). Furthermore, we proceeded to study the influence of miR-625-5p inhibition on CPSF7 expression in LINC00958 silenced cells. We observed that CPSF7 expression was down-regulated after knockdown of LINC00958, while increased again by miR-625-5p inhibitor, as illustrated in Fig. 4f and Additional file 3: Figure S3D. Together, these findings proved that LINC00958 served as a miR-625-5p sponge in the regulation of CPSF7.

### LINC00958 facilitates LAD cells proliferation, migration and invasion via up-regulating miR-625-5p-mediated CPSF7

**Fig. 5:**
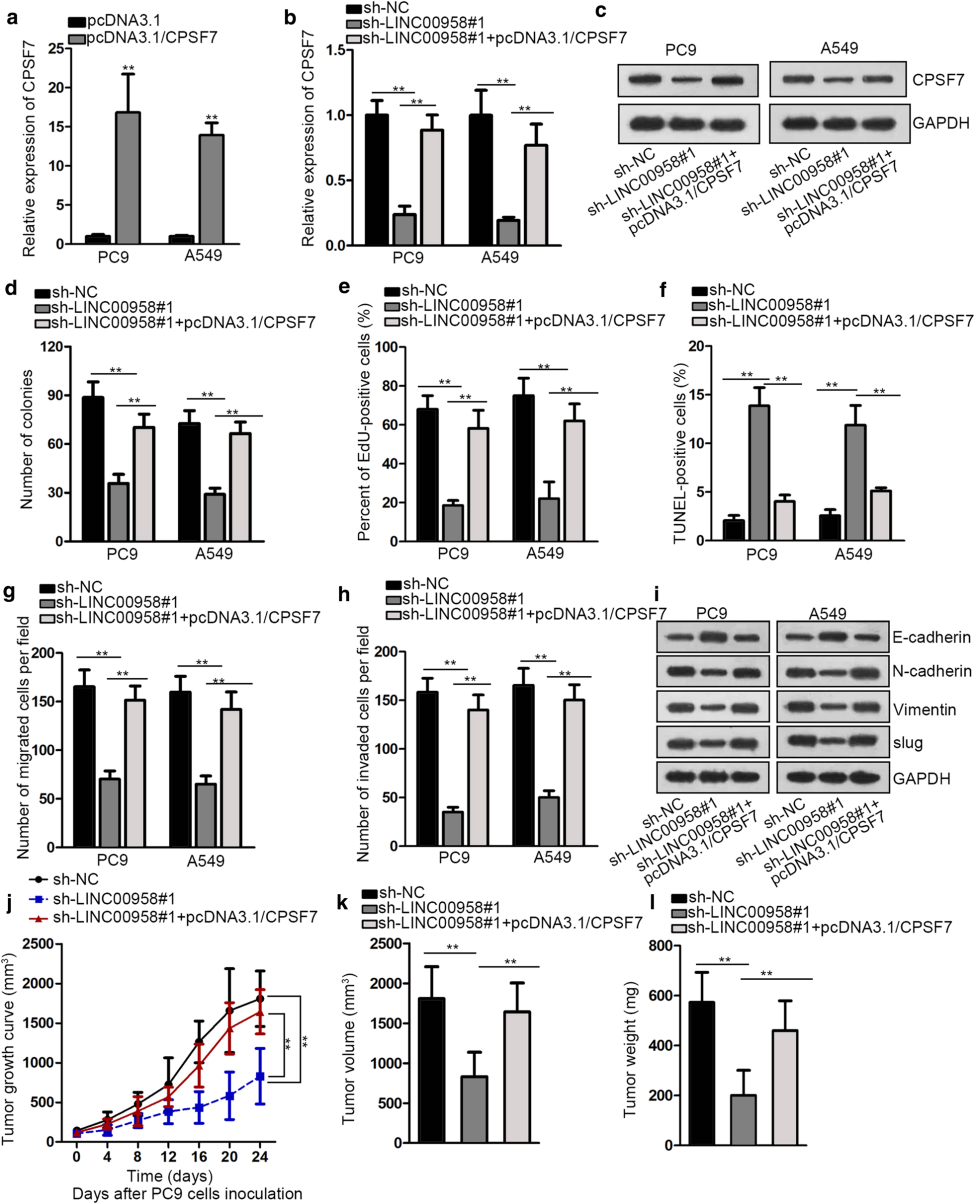
LINC00958 facilitates LAD cells proliferation, migration and invasion via up-regulating miR-625-5p-mediated CPSF7. **a** QRT-PCR assay was used to determine the transfection efficiency of pcDNA3.1/CPSF7. **b**, **c** QRT-PCR and western blot assays were used to determine the level of CPSF7 mRNA and protein. **d**–**i** A collection of rescue functional experiments were conducted among sh-NC, sh-LINC00958#1 and sh-LINC00958#1 + pcDNA3.1/CPSF7 groups. **j**–**l** Xenograft tumor growth curve, volume and weight were studied among sh-NC, sh-LINC00958#1 and sh-LINC00958#1 + pcDNA3.1/CPSF7 groups. **P < 0.01

To testify whether LINC00958 facilitates LAD by regulating miR-625-5p/CPSF7 axis, we performed a series of rescue assays. We firstly overexpressed CPSF7 by transfecting pcDNA3.1/CPSF7, as shown in Fig. 5a. Next, we transfected pcDNA3.1/CPSF7 into LINC00958 silenced PC9 and A549 cell lines. We observed that the expression of CPSF7 mRNA was notably diminished by LINC00958 knockdown, while increased again after overexpressing CPSF7 (Fig. 5b). The expression alternation of CPSF7 protein was found in line with its mRNA expression variation (Fig. 5c). CPSF7 overexpression could restore the proliferation ability inhibited by LINC00958 depletion (Fig. 5d, e). Moreover, elevating CPSF7 expression in PC9 and A549 cells could countervail the effects of LINC00958 deficiency on cell cycle and apoptosis (Additional file 3: Figure S3E, F). TUNEL assays further validated that the promoting impacts on apoptosis caused by silencing LINC00958 could be reversed by up-regulating CPSF7 expression (Fig. 5f). Furthermore, the suppressing effects on cell migration and invasion mediated by LINC00958 knockdown was abolished by overexpressing CPSF7 (Fig. 5g–i). To further explore the suppressing effect of LINC00958 knockdown on LAD progression, we established xenograft mice models by subcutaneously injecting transfected PC9 cells into nude mice. We found that LINC00958 knockdown could inhibit tumor growth and diminish tumor volume and weight, but then these effect was reversed by overexpressing CPSF7 (Fig. 5j–l). Besides, transfection with sh-LINC00958#1 remarkably lowered LINC00958 expression, but subsequent upregulation of CPSF7 exerted no significant impact on LINC00958 expression (Additional file 4: Figure S4A). Differently, elevating CPSF7 expression could recover the inhibitive effect of LINC00958 depletion on CPSF7 expression (Additional file 4: Figure S4B). Further, CPSF7 upregulation rescued the effect of silenced LINC00958 on the expression of proliferation-related proteins (Ki67, PCNA) and EMT-associated proteins (E-cadherin, N-cadherin) (Additional file 4: Figure S4C). Overall, these results further demonstrated thatLINC00958 mediates LAD progression through elevating CPSF7 expression.

## Discussion

LncRNAs have been extensively reported to exert regulatory effects in mRNA expression level by competing for the same combinable miRNAs [22]. The aberrantly expressed non-coding transcripts status is an important characteristic in cancer transcriptome [23]. Mounting researches have manifested that lncRNAs play crucial roles in the occurrence and progression of various cancers, such as breast cancer and prostate cancer [24, 25]. In this study, we observed the aberrantly elevated LINC00958 expression pattern in LAD. The inhibitory effects of LINC00958 knockdown on LAD cells proliferation, migration and invasion revealed the oncogenic properties of LINC00958 in LAD. This observation showed similarity to the reports of enriched present research on the oncogenic nature of LINC00958 in pancreatic cancer, gastric cancer (GC) and cervical cancer [26–28].

Transcription factor could promote or repress cancer progression via altering the expression profile of other genes. SP1 is a transcription factor that can activate or suppress transcription activity. We elucidated that up-regulation of LINC00958 in LAD was attributed to SP1 which served as a transcription activator of LINC00958 transcription in LAD. This finding proved some studies in which the transcription stimulation role of SP1 has been identified. For example, SP1 could interacted with lncRNA LINC00958 promoter region and facilitate its transcription [29]. This study demonstrated the critical role of transcription factor in the regulation of other genes and corresponding biological activities, which could provide implication for the research of abnormal expressed lncRNAs in carcinogenesis.

Increasing studies have manifested that lncRNAs could mediate the expression of the targets of miRNAs via regulating miRNA molecules, inevitably imposing an additional layer of post-transcriptional regulation [30]. In this study, we observed that miR-625-5p was notably down-regulated in LAD cell lines, and could be sponged by LINC00958. After determining the cytoplasmic location of LINC00958, we confirmed that LINC00958 served as a miR-625-5p sponge in LAD by mechanical experiments verification. MiR-625-5p inhibitor could counteract the anti-cancer effects induced by LINC00958 knockdown. This phenomenon proved that LINC00958 exhibited oncogenic property in LAD via down-regulating the expression of miR-625-5p and implicated that miR-625-5p was a crucial tumor suppressor gene in LAD development and progression. Identically, a previous report has detected the forced down-regulation of miR-625-5p could promote glioma aggressiveness progress [31]. Therefore, the aberrant exhibition of miR-625-5p in cancers might be a potential risking factor.

MiRNAs could affect the production of proteins by silencing target gene expression through recognition of 3′UTR sequences in target mRNAs [32]. We identified CPSF7 as the downstream target of miR-625-5p. In addition, LINC00958 could up-regulate the expression of CPSF7 via sponging miR-625-5p. CPSF7 overexpression counteracted the biological effects of silencing LINC00958 on LAD progression.

## Conclusion

We revealed the overexpression of LINC00958 in LAD cells, which was induced by the transcription factor SP1. Mechanically, LINC009581 elevated the expression of CPSF7 by acting as a miR-625-5p sponge, accelerating the development and progression of LAD. We proposed that LINC00958 might be utilized as a promising therapeutic target for LAD.

## Supplementary information

**Additional file 1: Figure S1.** A. LINC00958 expression in LAD tissues and matched non-tumor tissues was detected via RT-qPCR. B. The effect of LINC00958 knockdown on cell cycle was evaluated via flow cytometry. C. The expression of cycle-related proteins (cyclin D1, CDK4) and apoptosis-associated proteins (cleaved caspase-3, PARP) in different groups was detected via western blot. D-E. SP1 expression in LAD tissues and cells was analyzed via RT-qPCR. *P < 0.05, **P < 0.01.

**Additional file 2: Figure S2.** A. MiR-625-5p expression in LAD tissues and adjacent non-tumor tissues was examined via RT-qPCR. B. Cell cycle in PC9 and A549 cells transfected with different plasmids was analyzed via flow cytometry. C. Western blot analysis of cycle-related proteins (cyclin D1, CDK4) and apoptosis-associated proteins (cleaved caspase-3, PARP) was administrated in different groups. D-E. The original images of transwell migration and invasion assays in Fig. 3l, m. **P < 0.01.

**Additional file 3: Figure S3.** A. CPSF7 expression was detected via western blot analysis after miR-625-5p expression was elevated in PC9 cells. B-C. Upregulated expression of CPSF7 in LAD tissues and cells was observed via RT-qPCR and western blot, respectively. D. CPSF7 protein level was detected inn different groups via western blot analysis. E. Cell cycle in PC9 and A549 cells transfected with different plasmids was analyzed via flow cytometry. F. Western blot analysis of cycle-related proteins (cyclin D1, CDK4) and apoptosis-associated proteins (cleaved caspase-3, PARP) was administrated in different groups. **P < 0.01.

**Additional file 4: Figure S4.** A-B. The expression of LINC00958 and CPSF7 in different groups was detected via qRT-PCR. C. IHC analysis of proliferation-related proteins (Ki67, PCNA) and EMT-associated proteins (E-cadherin, N-cadherin) was conducted in different groups. **P < 0.01.

## Data Availability

Experimental data and materials are not shared.

## Abbreviations

lncRNAs: long non-coding RNAs
LAD: lung adenocarcinoma
ceRNA: competing endogenous RNA
qRT-PCR: quantitative real-time PCR
ChIP: chromatin immunoprecipitation
FISH: fluorescence in situ hybridization
RIP: RNA immunoprecipitation
NSCLC: non-small cell lung cancer
TSS: transcription start site
GC: gastric cancer
