# Supplementary figures and images for: SP1 induced long non-coding RNA LINC00958 overexpression facilitate cell proliferation, migration and invasion in lung adenocarcinoma via mediating miR-625-5p/CPSF7 axis
Source: Cancer Cell Int. 2020 Jan 23;20:24. doi: 10.1186/s12935-020-1099-0 (PMC6979366; doi:10.1186/s12935-020-1099-0)

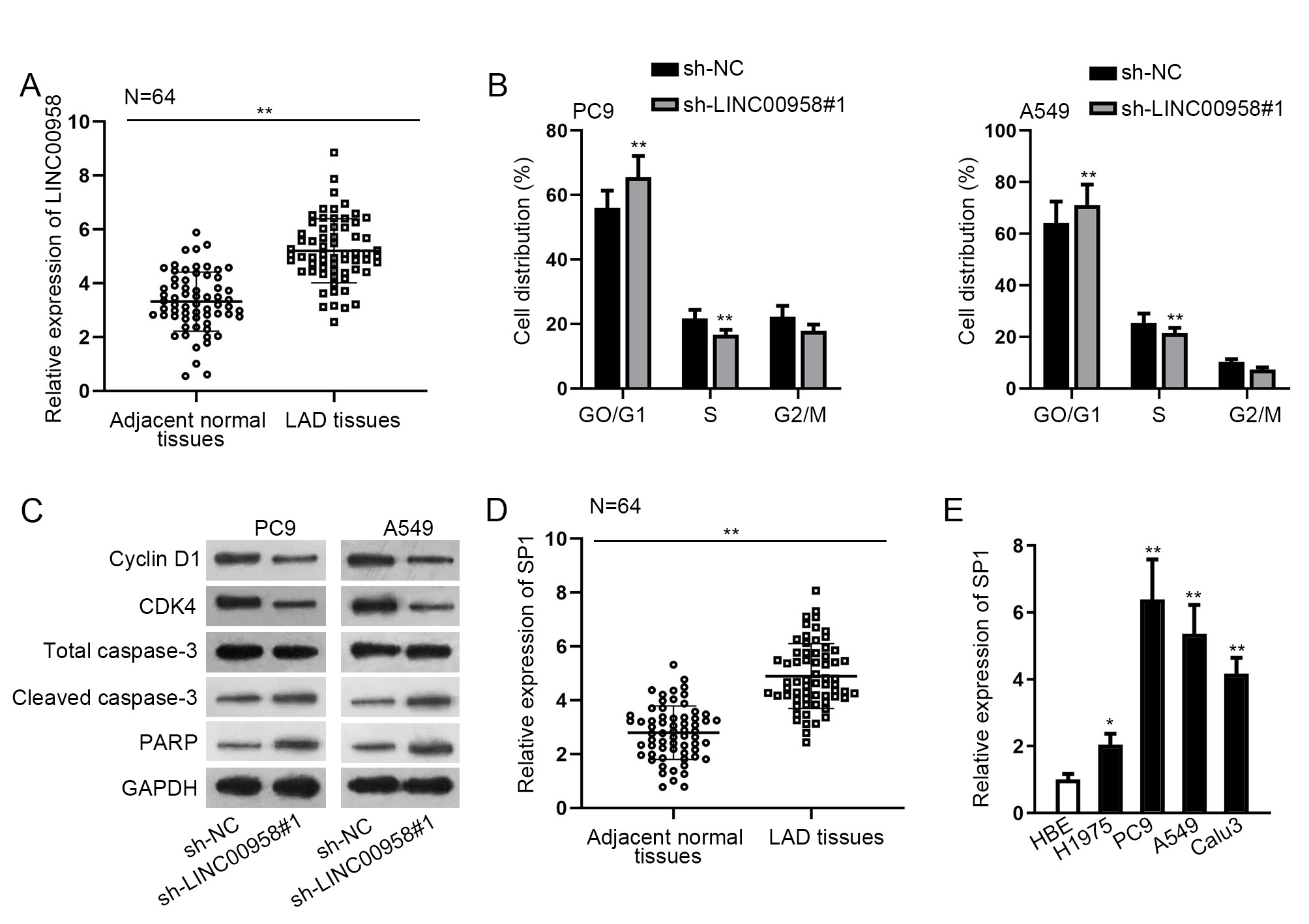

Supplement: Supplementary file 1 — Additional file 1: Figure S1. A. LINC00958 expression in LAD tissues and matched non-tumor tissues was detected via RT-qPCR. B. The effect of LINC00958 knockdown on cell cycle was evaluated via flow cytometry. C. The expression of cycle-related proteins (cyclin D1, CDK4) and apoptosis-associated proteins (cleaved caspase-3, PARP) in different groups was detected via western blot. D-E. SP1 expression in LAD tissues and cells was analyzed via RT-qPCR. *P < 0.05, **P < 0.01. [file 12935_2020_1099_MOESM1_ESM.jpg]

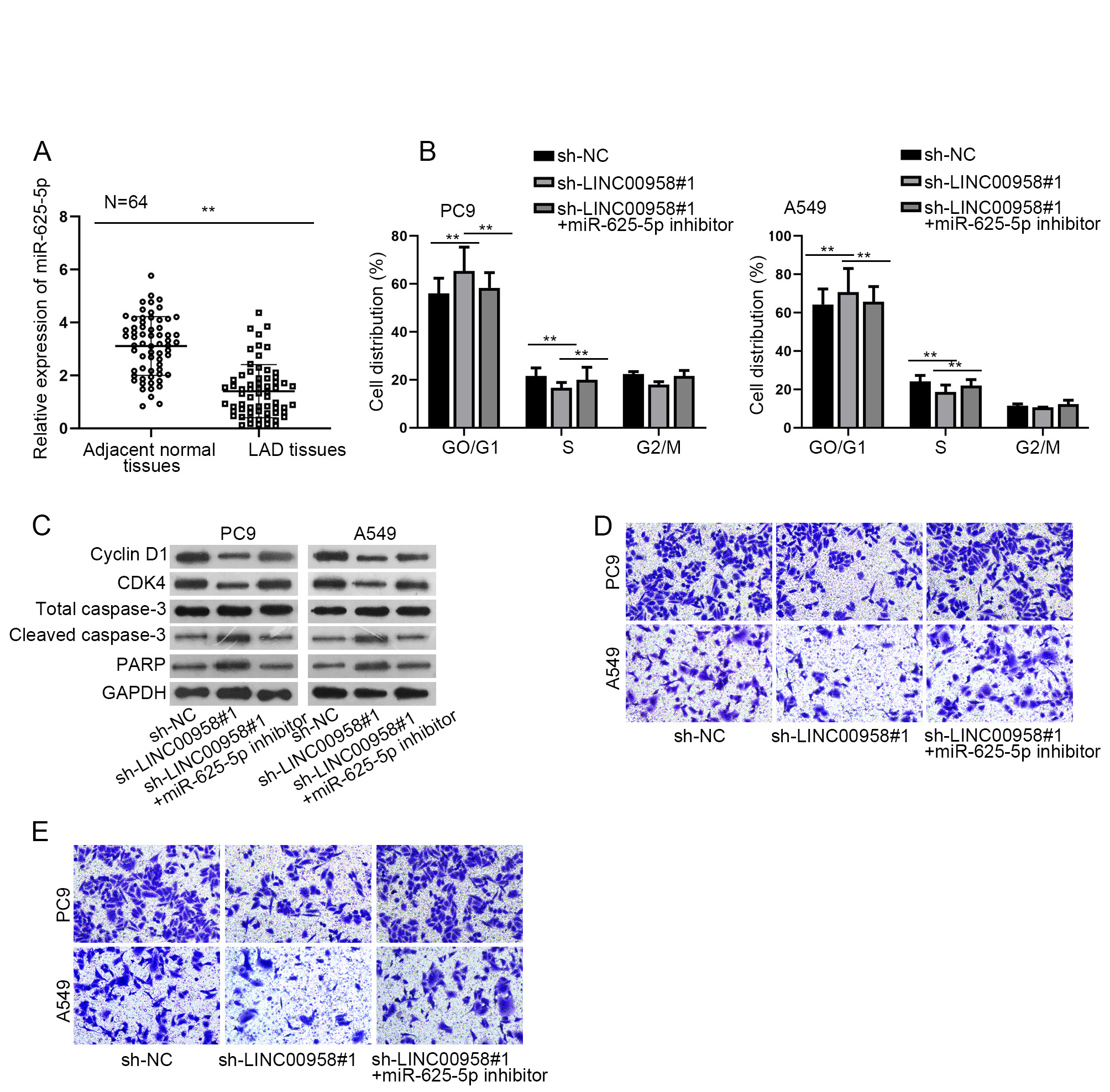

Supplement: Supplementary file 2 — Additional file 2: Figure S2. A. MiR-625-5p expression in LAD tissues and adjacent non-tumor tissues was examined via RT-qPCR. B. Cell cycle in PC9 and A549 cells transfected with different plasmids was analyzed via flow cytometry. C. Western blot analysis of cycle-related proteins (cyclin D1, CDK4) and apoptosis-associated proteins (cleaved caspase-3, PARP) was administrated in different groups. D-E. The original images of transwell migration and invasion assays in Fig. 3l, m. **P < 0.01. [file 12935_2020_1099_MOESM2_ESM.jpg]

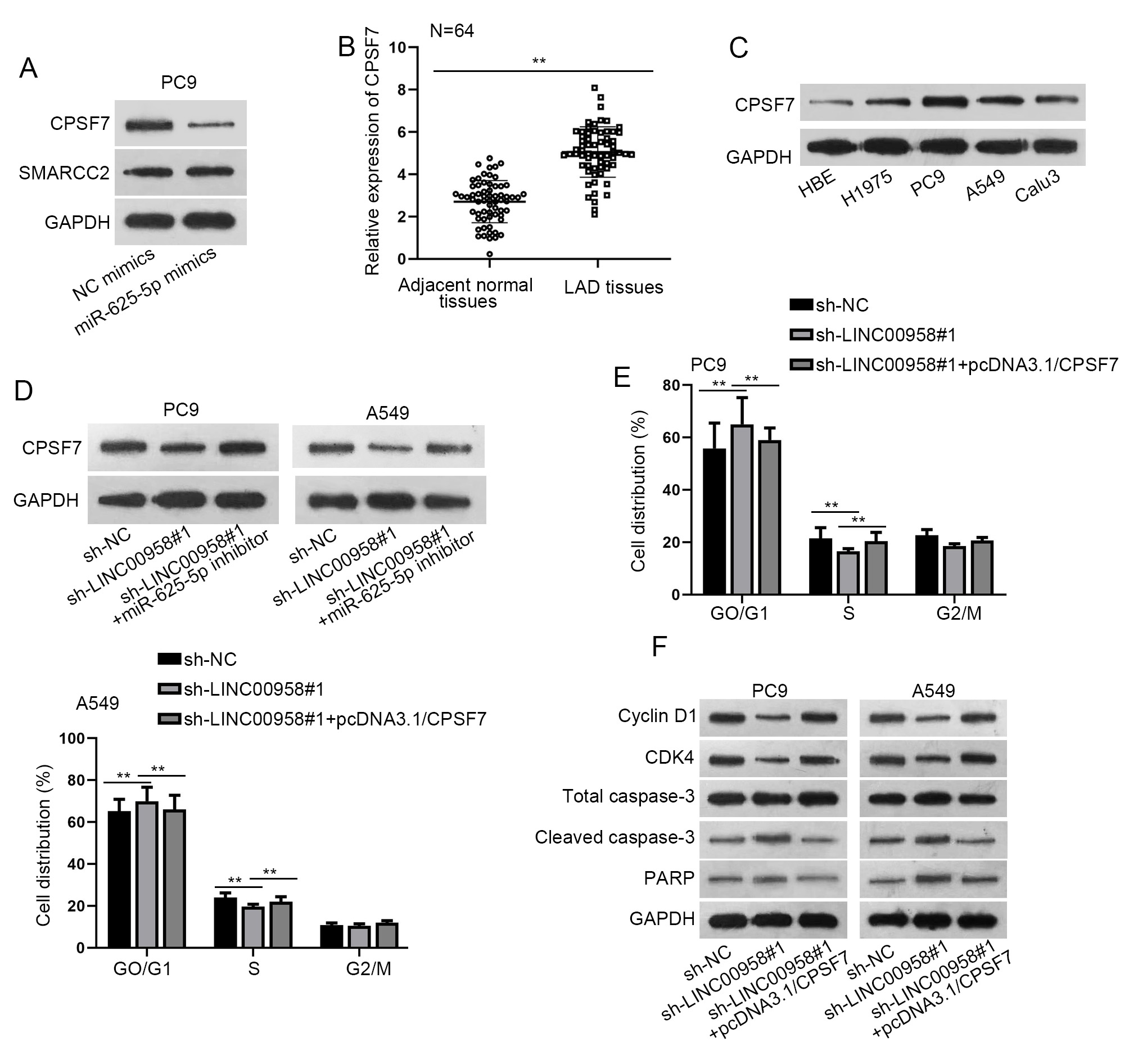

Supplement: Supplementary file 3 — Additional file 3: Figure S3. A. CPSF7 expression was detected via western blot analysis after miR-625-5p expression was elevated in PC9 cells. B-C. Upregulated expression of CPSF7 in LAD tissues and cells was observed via RT-qPCR and western blot, respectively. D. CPSF7 protein level was detected inn different groups via western blot analysis. E. Cell cycle in PC9 and A549 cells transfected with different plasmids was analyzed via flow cytometry. F. Western blot analysis of cycle-related proteins (cyclin D1, CDK4) and apoptosis-associated proteins (cleaved caspase-3, PARP) was administrated in different groups. **P < 0.01. [file 12935_2020_1099_MOESM3_ESM.jpg]

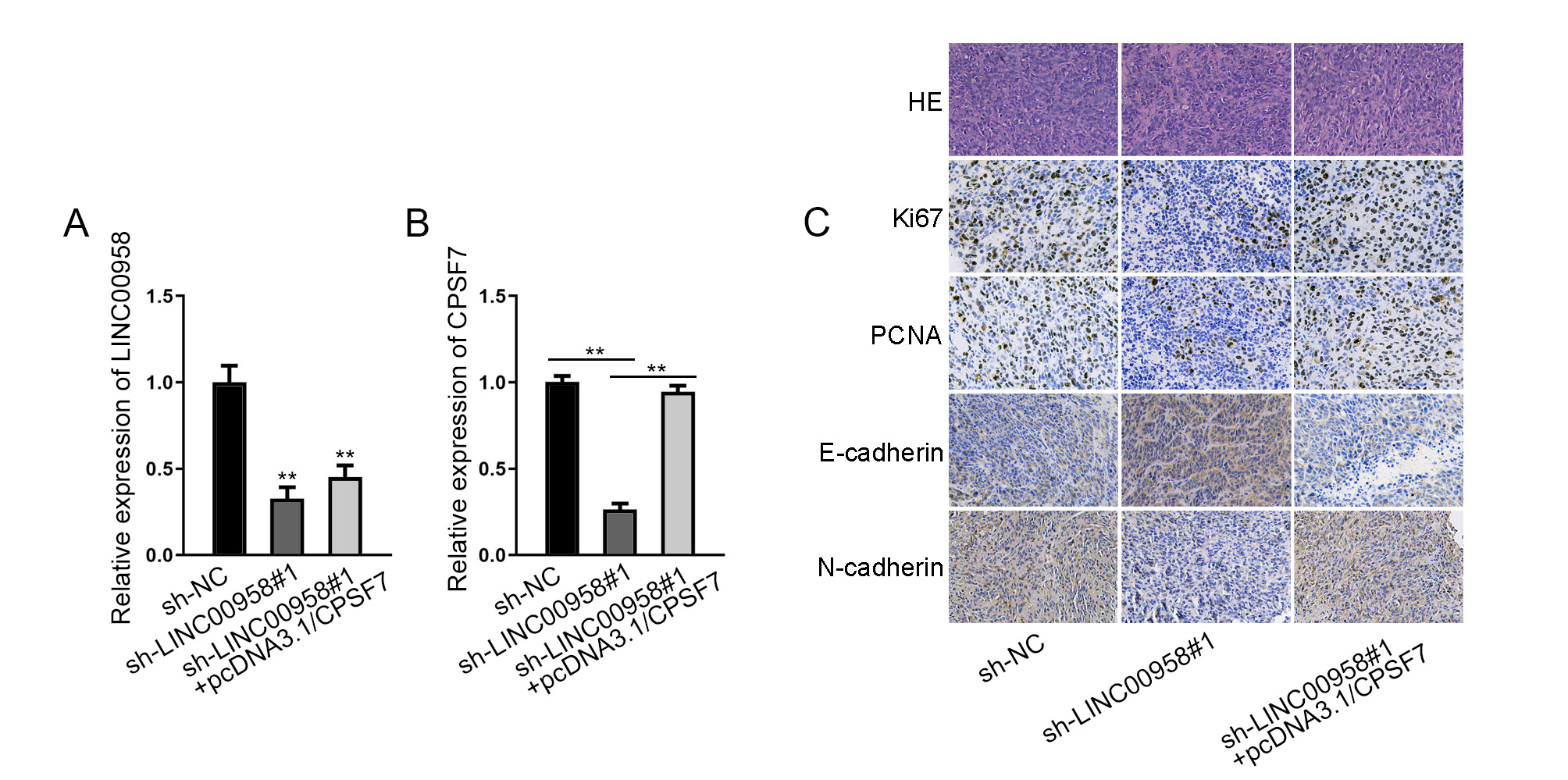

Supplement: Supplementary file 4 — Additional file 4: Figure S4. A-B. The expression of LINC00958 and CPSF7 in different groups was detected via qRT-PCR. C. IHC analysis of proliferation-related proteins (Ki67, PCNA) and EMT-associated proteins (E-cadherin, N-cadherin) was conducted in different groups. **P < 0.01. [file 12935_2020_1099_MOESM4_ESM.jpg]
